# Effectiveness of the YourCall™ text message intervention to reduce harmful drinking in patients discharged from trauma wards: protocol for a randomised controlled trial

**DOI:** 10.1186/s12889-016-3967-z

**Published:** 2017-01-09

**Authors:** Shanthi Ameratunga, Bridget Kool, Sarah Sharpe, Papaarangi Reid, Arier Lee, Ian Civil, Gordon Smith, Vanessa Thornton, Matthew Walker, Robyn Whittaker

**Affiliations:** 1 Section of Epidemiology & Biostatistics, School of Population Health, University of Auckland, Private Bag 92019, Auckland Mail Centre 1142, Auckland, New Zealand; 2 Te Kupenga Hauora Māori, Faculty of Medical & Health Sciences, University of Auckland, Private Bag 92019, Auckland Mail Centre 1142, Auckland, New Zealand; 3 Department of Surgery, Auckland City Hospital, 2 Park Road, Grafton, Auckland, 1023 New Zealand; 4 West Virginia University School of Public Health, 1 Medical Center Drive, PO Box 9190, Morgantown, WV 26506-9190 USA; 5 Middlemore Hospital, Private Bag 93311, Otahuhu, Auckland, 1640 New Zealand; 6 North Shore Hospital, Shakespeare Road, Takapuna, Auckland, 0622 New Zealand; 7 National Institute for Health Innovation, School of Population Health, University of Auckland, Private Bag 92019, Auckland Mail Centre 1142, Auckland, New Zealand

**Keywords:** Alcohol use, Text messaging, Wounds and injuries, Brief intervention, Motivational interviewing, Trauma centers, Mobile health

## Abstract

**Background:**

Behavioural brief interventions (BI) can support people to reduce harmful drinking but multiple barriers impede the delivery and equitable access to these. To address this challenge, we developed YourCall™, a novel short message service (SMS) text message intervention incorporating BI principles. This protocol describes a trial evaluating the effectiveness of YourCall™ (compared to usual care) in reducing hazardous drinking and alcohol related harm among injured adults who received in-patient care.

**Methods/design:**

Participants recruited to this single-blind randomised controlled trial comprised patients aged 16-69 years in three trauma-admitting hospitals in Auckland, New Zealand. Those who screened positive for moderately hazardous drinking were randomly assigned by computer to usual care (control group) or the intervention. The latter comprised 16 informational and motivational text messages delivered using an automated system over the four weeks following discharge. The primary outcome is the difference in mean AUDIT-C score between the intervention and control groups at 3 months, with the maintenance of the effect examined at 6 and 12 months follow-up. Secondary outcomes comprised the health and social impacts of heavy drinking ascertained through a web-survey at 12 months, and further injuries identified through probabilistic linkage to national databases on accident insurance, hospital discharges, and mortality. Research staff evaluating outcomes were blinded to allocation. Intention-to-treat analyses will include assessment of interactions based on ethnicity (Māori compared with non-Māori).

**Discussion:**

If found to be effective, this mobile health strategy has the potential to overcome current barriers to implementing equitably accessible interventions that can reduce harmful drinking.

**Trial registration:**

Universal Trial Number (UTN) U1111-1134-0028. ACTRN12612001220853. Submitted 8 November 2012 (date of enrolment of first participant); Version 1 registration confirmed 19 November 2012. Retrospectively registered.

## Background

Alcohol misuse is a leading cause of premature death and disability in New Zealand incurring substantial costs to society and health services [1]. A multi-country study found the proportion of Emergency Department visits associated with alcohol use to be particularly high in New Zealand [2]. Injury is the largest contributor to alcohol-related mortality [1, 3] and alcohol is widely acknowledged as the leading risk factor for injury [1, 3]. Can a mobile health intervention reduce the recognised excess risk of ongoing harm and injury recurrence [4, 5] experienced by patients with hazardous drinking patterns discharged from trauma wards?

The imperatives to scale up population-based strategies to reduce alcohol-related harm are obvious. The most effective among these include increasing the minimum purchasing price and age, reducing availability and access, and restricting advertising and other promotions of alcohol [6, 7]. However, these efforts are challenged directly and indirectly by the industry with capitulation to commercial interests a high risk in many countries [8]. Targeted interventions in clinical settings as a complementary approach can, at least partly, mitigate risks of on-going harm in contexts where the prevalence of alcohol problems is high.

The World Health Organization, the U.S. Substance Abuse and Mental Health Services Administration, and the U.S. Preventive Services Task Force, among other agencies advocate point-of-care opportunistic screening and brief interventions (BIs) to reduce risks of on-going harm [9–11]. The U.S. National Commission on Prevention Priorities ranks this approach to be among the five most effective clinical preventive services [12]. Screening and BIs for harm aim to identify a real or potential alcohol use problem and motivate an individual to lower their risk for alcohol-related problems [13, 14]. Drawing on a variety of behaviour change theories, these interventions employ three common strategies: 1) giving information or feedback, 2) understanding patients’ views of drinking and enhancing motivation, and 3) giving advice and negotiating responsive behaviour change [14].

A robust body of research indicates that screening and BIs in trauma care settings can reduce alcohol intake and lower levels of hospital re-attendance [6, 15]. However, such interventions are infrequently implemented in New Zealand [16]. In the U.S., despite screening and interventions for alcohol-related disorders being required for trauma centre accreditation [10] with related recommendations in the U.S. Trauma Service Guidelines [14], many centres fall short of the intended goals [17–19]. A 2011 U.S. state-based behavioural risk factor survey found fewer than one in six U.S. adults report ever discussing alcohol use with a doctor or other health professional; and two-thirds (65.1%) of those who report binge drinking 10 times or more in the previous month deny ever having this conversation [20].

Barriers to screening and intervention commonly proffered by health professionals include the lack of time, resources, training and workforce capacity; self-efficacy; discomfort discussing the topic; perceived difficulties interacting with patients who use substances; anticipated patient resistance; and scepticism about the likely benefits [16–19, 21]. Regardless of the explanation, the deficiencies encountered correspond to an important gap in quality of care. This is particularly unfortunate as trauma patients indicate they want healthcare teams to address their problems with alcohol [18, 22]. Furthermore, unlike many other harms with longer lead times, injury risks respond relatively rapidly to changes in drinking. However, the half-life of ‘teachable moments’ may be short and the risks of inequitable, delayed or poor access to standard referral pathways for alcohol misuse problems are high [23].

Social determinants can strongly influence alcohol use with more impoverished groups at significantly greater risk of experiencing related harms [24–26]. This is of particular concern in New Zealand, where Māori (indigenous people) have disproportionately high rates of injury and less access to health and rehabilitation services compared with non-Māori [27]. These are symbolic of significant breaches of the principles of partnership, participation and protection that underpin the relationship between the Government and Māori with respect to the founding document of Aotearoa New Zealand, Te Tiriti o Waitangi (The Treaty of Waitangi).

YourCall™ is a novel SMS-text message intervention designed to address the challenges noted above. The overall goal is to reduce hazardous drinking and alcohol-related harm among adults admitted to hospital following an injury and facilitate equitable benefits for Māori relative to non-Māori.

### Rationale for the intervention mode

It is argued that ‘mHealth (mobile health) technologies have the potential to change every aspect of the health care environment and to do so while delivering better outcomes and substantially lowering costs’ [28]. The vast majority of mHealth interventions tested to date have focused on chronic care management (e.g., diabetes and asthma), and health behaviours such as physical activity, obesity prevention and smoking cessation [28, 29].

To achieve our intended goal of supporting the reduction of hazardous drinking, we developed the YourCall™ intervention drawing on the extensive experience of members of the research team in designing and evaluating a range of mHealth interventions [30–34]. We commenced the process with a robust consultation process and feasibility study affirming the interest and acceptability of this approach among trauma patients [23]. As published in this journal previously [35], we then used a formal intervention development format to design, pre-test and refine a tailored suite of 16 text messages to be delivered over a four-week period after injury patients were discharged from hospital. The intervention development team included injury prevention practitioners, Māori and Pacific researchers, emergency and trauma service providers, substance use treatment providers, community stakeholders, and experts in mobile health technology. Our previous publication provides detailed information on the consultation process and feedback, the underpinning theory, development and final content of the intervention [35].

This paper describes the study design, baseline and outcome data collection procedures, and approach to analyses in the single-blind randomised controlled trial evaluating the effectiveness of the YourCall™ intervention in Auckland, New Zealand. The project is ongoing with the current stage involving collection of the final phase of secondary outcome data, and the analysis and presentation of the primary outcome data.

## Methods/design

### Design and hypothesis

**Fig. 1:**
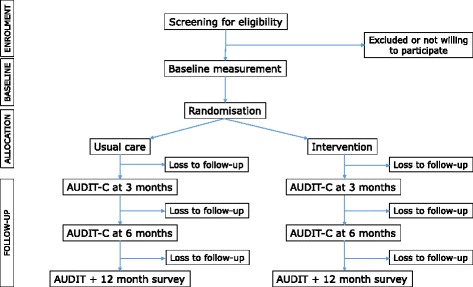
CONSORT diagram for YourCall™ trial

The YourCall™ study is a two-group, parallel, single-blind randomised controlled-trial. Individuals who screened positive for hazardous drinking (Alcohol Use Disorders Identification Test [AUDIT] score 7-15 for females, 8-15 for males) were individually randomised to an intervention group or a usual care group (control) in a 1:1 allocation ratio (Fig. 1). We hypothesise that in comparison to hazardous drinkers discharged following an injury admission who received usual care (control group), those who received a structured sequence of SMS-text messages (YourCall™) incorporating brief intervention from harm principles:

1. Will have reduced hazardous drinking at 3, 6 and 12 months follow-up as assessed by the AUDIT-C score (primary outcome at 3 months)
2. Will have fewer subsequent medically attended injuries (identified through record linkage to national databases), and
3. Will experience less adverse health and social outcomes, e.g., sexual harm, mental health problems, and legal consequences of heavy drinking (identified through a self-report survey at 12-months follow-up).

We also hypothesise that the effectiveness of the intervention (as measured above) will be similar among Māori and non-Māori.

### Participant recruitment and eligibility criteria

Participants to this study were recruited from November 8, 2012 to December 12, 2013, from one of the three trauma admitting hospitals (Middlemore, North Shore and Auckland City hospitals) in Auckland, New Zealand. The region includes urban, suburban and rural areas and has a population of approximately 1.4 million [36].

Trial eligibility criteria were (i) patients aged 16 to 69 years admitted to one of the three recruiting hospitals with an injury, (ii) screened positive for hazardous alcohol use (AUDIT score: 7-15 for females, 8-15 for males), (iii) used a mobile phone which is for their own use (i.e., not shared with another), (iv) were willing and able to read and send text messages, (v) were discharged home, (vi) were able to complete surveys in English, (vii) resided in New Zealand for the duration of the trial, and (viii) were competent and willing to provide informed consent to take part in the trial. Patients who were pregnant or whose injury was as a result of self-harm were excluded from this trial.

### Intervention

Participants in the intervention group received the active intervention through an automated system delivering one text message approximately every two days for 4 weeks (16 messages in total). Messaging was designed to commence on a Monday and finish on a Saturday with week-day messages sent at 7 pm and weekend messages at 3 pm. No specific training was provided in how to access or interpret messages. All participants were provided *The straight up guide to standard drinks* brochure [37]. This provided information about the alcohol content of different alcohol drinks as well as contact details for the Alcohol Drug Helpline, a telephone-based free and confidential service funded by the Health Promotion Agency and the Ministry of Health (www.alcoholdrughelp.org.nz).

#### Intervention development and delivery

We iteratively designed, developed, and tested the intervention using a formal process to integrate existing evidence with the input from clinical staff, patients with injury, and Māori and Pacific reference groups [35]. As part of this process, we developed a bank of SMS-text messages underpinned by BI principles and behaviour change theory. The content of messages provided participants with feedback about their drinking, encouraged contemplation about their drinking, recommended cutting down on their drinking, provided information and strategies to assist reducing alcohol consumption, and support and encouragement to aid this. The SMS text-messages used in the intervention were developed and tested in English, and translated into Te Reo Māori (the language of the indigenous people). The text message intervention had three language-based pathways for people to choose between: 1) English text messages with Te Reo Māori words of welcome and encouragement, 2) Te Reo Māori text messages, and 3) English text messages with an option to receive a greeting in Samoan, Tongan, Cook Island Māori, Niuean, Tokelauan, Tuvaluan, or Fijian. The intervention text messages have been previously published [35].

#### Comparator: Usual care

Consistent with similar trials in trauma settings and discussions with the advisory group and clinical staff, the most appropriate comparator was deemed the provision of usual care by the admitting hospital. Participants allocated to the usual care group received just one initial message: ‘Hi, thanks 4 taking part in the study. We will txt u with some questions in 3 months time’. As noted earlier, all participants received a copy of the brochure described previously. No other concomitant care was provided, and we did not specify any interventions as permitted or prohibited.

#### Procedures promoting intervention fidelity and reducing contamination

With a view to promoting engagement in the trial and as an acknowledgement of participation, participants were provided with a NZ$20 supermarket voucher at the time of recruitment. In order to ensure the intervention delivered was as close to what could be expected from a service in the field, we did not implement specific strategies to monitor adherence to the intervention.

Research assistants responsible for the recruitment of participants and trial participants were advised that not everyone will be receiving the exact same number or type of messages. In order to reduce between-group contamination, SMS text message content was stored remotely in a message bank on a secure server. An automated delivery system was used to send SMS text-messages at no cost to participants. The server and delivery system were managed by technicians with no direct contact with trial participants.

### Outcomes

#### Primary outcome measures

The primary clinical outcome is the difference in hazardous alcohol use, assessed using the pre-validated AUDIT-C [38] tool (a shorter version of the AUDIT), between intervention and control groups at 3 months. The AUDIT-C is scored on a scale of 0–12 (scores of 0 reflect no alcohol use). In men, a score of 4 or more is considered positive for problem alcohol use; in women, a score of 3 or more is considered positive. Generally, the higher the AUDIT-C score, the more likely it is that the patient’s drinking is affecting their health and safety.

#### Secondary outcomes

Self-reported data were collected on health and social outcomes and legal consequences of heavy drinking at the 12-month follow-up period using a web-based questionnaire (or telephone-based for those without web access).

Medically attended injuries during the follow-up period will be identified by probabilistic record linkage to claims lodged with the Accident Compensation Corporation (ACC: New Zealand’s national no-fault accident insurance scheme), hospital discharge and mortality databases using unique identifiers (the National Health Index number).

### Trial procedures

#### Baseline data collection

Participants were recruited from patients admitted to one of the three recruiting hospitals for an injury-related cause. All those who were potentially eligible were given information (verbal and written) about the trial. If they were interested in participating, verbal consent was gained to determine eligibility (the form used ascertained the age, gender and ethnicity of respondents) and the initial screening undertaken i.e. alcohol drinker, mobile phone user, willing to receive and send SMS messages, able to complete questionnaires in English, not pregnant, and a New Zealand resident. Written informed consent was obtained from those meeting the eligibility criteria. These people were then screened using the AUDIT tool. Trained research assistants used *The straight up guide to standard drinks* brochure [37] to assist with the AUDIT tool questions. Participants screened as having non-hazardous drinking patterns (AUDIT score <7 for females, <8 for males) were excluded from randomisation. Participants with AUDIT scores >15 indicating possible alcohol dependence were also excluded from randomisation, provided with the number of the national Alcohol Helpline and the local community alcohol and drug services, and offered referral to that service.

Research assistants were present at the hospital every day of the week during regular working hours for the duration of the recruitment period to ensure participant enrolment was maximised. In addition, members of the clinical team informed potentially eligible patients that the study was taking place and that they may be contacted by one of the research team members. Trauma coordinators based at the three hospitals would also advise the research assistants if potentially eligible patients had been admitted. Hospital case-mix reports were generated on a regular basis as a method of double checking that all potential eligible patients had been approached.

Baseline data collected from study participants included: demographic details (age, gender, ethnicity), contact details (residential address, phone numbers, email address), mobile phone details and patterns of use, cigarette and drug use, the role of alcohol in this injury, and employment and education information. Participants were then given the choice of selecting one of the three language-based pathways through which the text messages are transmitted.

In accordance with the informed consent provided by participants, research assistants extracted details pertaining to the participant’s admission from their medical record (e.g. date of injury, date and time of admission and discharge, mechanism and intent of injury, blood alcohol level, and nature of injuries).

#### Allocation - Randomisation

Individuals screening positive for hazardous drinking (AUDIT score 7-15 for females and 8-15 for males) were randomised using a secure, remote, web-based computer schedule at the time of discharge from hospital. Computer-based randomisation ensured balance for factors such as age, gender, ethnicity and recruitment hospital, and the study procedures established ensure adherence to allocation concealment.

In this single blind trial, the researchers, members of the trial Steering Committee, Clinical Reference Group, Management Committee, and the data management group (National Institute for Health Innovation [NIHI]) remained blinded to treatment allocation until the code was broken (after the last follow-up visit was completed). All baseline data collection was undertaken by research assistants who were blind to treatment allocation. Primary outcome data collection was automated and obtained via text message. Secondary outcome data were collected using an on-line survey (the latter was administered by telephone if on-line completion was not an option) or through anonymised data linkage.

#### Follow-up

Follow-up self-report surveys at 3 and 6, months using the AUDIT-C tool were conducted via text message. Three text-backs were required at 3 and 6 months follow-up. At the 9 month follow-up time point participants were asked via SMS messaging to text back to confirm their email address (which was provided at baseline). The total number of text-backs required was 7 text messages and participants were aware that they bear the cost involved. For participants on pre-pay plans this would cost a maximum of NZ$1.40. In order to acknowledge this cost and their participation in the study, treatment and control group participants receive a shopping voucher at the time of recruitment.

The 12-month web-based survey developed in LimeSurvey (open source software) included the full 10-item AUDIT as well as questions to gather self-reported data on health and social outcomes and legal consequences of heavy drinking during the follow-up period. Participants were asked two sets of ‘drinking consequences’ drawn from the GENACIS project (http://www.genacis.org/11) [39]. These comprised seven questions relating to possible alcohol-related ‘harms’ in the previous 12 months and seven questions relating to possible alcohol-related ‘troubles’. They were also asked questions about their alcohol-related healthcare service seeking behaviours, their current feeling about their readiness to change using a visual analogue scale [40], their experience with hangovers, and their experience of being in the study (i.e. the ‘good things’ about being in the study and what they ‘liked least’). Participants who were unable to complete the web-survey or preferred an alternative, had the survey administered by phone.

To encourage participants to reply to text message questions at the 3, 6, and 9 month follow-up points and to complete the online survey at 12 months, an incentive was offered. As part of the text message communication at each of the follow-up time points, participants were advised they would be eligible for a prize draw for a $200 supermarket voucher. Once each follow-up period had been completed for all participants, one participant was selected randomly from all those who had completed follow-up for that time-point. Four different participants each received a voucher.

Information regarding injuries during follow-up will be obtained via probabilistic record linkage to national databases on injury-related insurance claims (Accident Compensation Corporation) and Ministry of Health data on hospital discharges, ambulatory care visits and deaths at 24 months following the index admission. In general, approximately 24 months following an event is required to assure completeness of injury data recorded in routine databases with confirmed cause of death information for fatal injury events.

#### Screening of text-backs from participants

A specification was built into the intervention content delivery system to identify text responses from participants indicating they wish to stop receiving text messages. On receipt of such a message, an automated response acknowledging receipt of the participant’s message was sent and then all subsequent text messages were automatically stopped. Participants who requested to be withdrawn from the study had no further data collected.

Participants who sent an unsolicited text-back were sent an automated text message as follows: ‘From YourCall: This is an automated reply to your txt. If u are in an emergency situation, call 111’.

In addition to the above, a daily report was generated detailing any unrecognised responses received from participants. This was reviewed by a dedicated study team member who determined the appropriate level of action, the details of which were recorded in the ‘Text Response Register’. The levels of action included:

- No action required (e.g. conversational or acknowledgement text-backs)
- Action required on a study-related matter (e.g. participant asking to stop messages in a way that was not recognised by the computer system)
- Action required on another matter (e.g. message indicating personal distress; response was determined on a case-by-case basis and reviewed by the Principal Investigator

### Sample size calculations

Based on the published literature on distribution in AUDIT-C scores in previous trials, we aimed to recruit approximately 570 participants to this study. This was based on a conservative estimate that 25% of patients screened will have AUDIT scores in the eligibility range for moderately hazardous drinking and a 75% participation rate. We anticipated that 400 (70%) of these participants would complete 12 months of follow-up. This sample size provides 80% power to detect a true difference of 7.5% (i.e., a mean difference between groups in AUDIT-C scores of 0.51) at the 0.05 level of significance.

As designed, this is a large trial relative to most alcohol intervention studies conducted previously. By recruiting patients from all major trauma-admitting hospitals in the greater Auckland region, we expected to recruit sufficient participants to determine effects on alcohol misuse and related harms (including injury recurrence) with adequate statistical power in a resource efficient manner.

The study also aimed to recruit as many Māori participants as possible with a minimum of 20% of the total sample, a situation deemed feasible given the ethnic distribution in the Auckland region. This sample size was expected to provide good power to test consistency in effect for Māori compared with non-Māori.

### Data collection and management

The study database was constructed in Oracle. Participant data was collected by research assistants using password protected iPads. Case report form (CRF) data are imputed and validated using eCRFs screens on the study website. Validation rules for each CRF were specified by the study manager, in association with the NIHI data manager. These rules included range checks so that inaccuracies in data collection could be identified early. A query was raised as soon as any values entered were outside the allowed range or if data were missing. As soon as a query was raised, the research assistants who collected and entered the data resolved the issue and amended the electronic CRFs. The management of all databases associated with this trial was undertaken by the data management and information technology groups at the NIHI. The database was regularly backed up and password-protected, with differing levels of access for different research staff depending on their roles and responsibilities.

### Study monitoring and quality control

The Study Steering Committee was responsible for the design of the study, recruitment and training of research assistants, development of the analysis plan, analysis and write of the study, and the dissemination of study findings. The members of this committee included: SA (principal investigator), RW (named investigator [NI]), BK (NI, project manager), PR (NI), GS (NI), MW (NI), VT (NI), IC (NI), SS (NI, lead researcher, project coordinator).

The coordinating centre, under the guidance of the Study Steering Committee was responsible for the overall coordination of the study including: the preparation and distribution of study materials, investigator meetings, registration and randomisation services (undertaken by NIHI), data management, achievement of recruitment targets, data collection and processing (in conjunction with NIHI), developing the intervention delivery system (automated text messages), adherence to the study protocol, statistical analysis, data quality, regulatory reporting requirements (ethics, funders), and the dissemination of findings from the study. The team included the following personnel: the project manager (BK), the project coordinator (SS), a data manager, a senior IT developer, a biostatistician, and a data services specialist.

Based on an assessment by the Health Research Council of New Zealand when the study was funded, the YourCall™ trial was deemed to not require an independent Data Monitoring Core Committee [41]. An external independent trial monitor was responsible for implementing the procedures documented in a Study Monitoring Manual. Each recruitment site and the coordinating centre was monitored to ensure that trial site staff conducted, recorded, and reported the trial according to the Protocol, the Manual of Procedures and The International Conference on Harmonisation of Technical Requirements for Registration of Pharmaceuticals for Human Use Good Clinical Practice guidelines [42]. Monitoring visits provided an opportunity for training, for discussion of trial issues, and for the establishment of good working relationships between trial personnel.

This study did not have an ‘endpoint adjudication committee’. No interim analyses were planned and the secondary injury outcomes were obtained after these were collated in routinely collected data in national databases.

### Reporting of adverse events for this trial

Adverse events will be reported and categorised with respect to their likely relationship to the intervention (i.e., definitely, possibly, not related). Adverse events that might be reasonably related to SMS text messaging include hand or finger pain, or involvement in an accident as a result of sending or receiving a text relating to the study. No specific provisions were made for ancillary or post-trial care, or for compensation to those who suffer harm from trial participation. New Zealand’s accident compensation scheme provides 24-hour no-fault personal accident insurance cover.

### Statistical methods

Statistical analyses of the data gathered in this trial are being performed using SAS version 9.4 (SAS Institute Inc. Cary NC). All participants considered for eligibility for the study will be accounted for in analyses. The principal evaluations of interest in this study subscribe to the ‘intention to treat’ principle, i.e. all participants are analysed in the group to which they were randomised, regardless of whether they discontinued or deviated from the protocol. However, no outcome data were collected from participants who withdrew, beyond their withdrawal day.

This trial did not include plans for interim analyses or stopping guidelines. Accordingly, analyses are being performed after 12 month follow-up has been completed. Secondary outcome data regarding subsequent injury events are expected to require a minimum of 24 months to lapse from recruitment given the time required to get cause of death information in national mortality databases. Missing data are not imputed. All statistical tests are two-tailed with a 5% significance level maintained throughout the analyses.

A CONSORT trial profile will summarise the number of participants who were considered, fulfilled eligibility criteria, reasons for exclusions, randomised, withdrawn, and lost to follow-up. Data for the intervention and control groups are being summarised with respect to baseline demographic variables (age, sex, ethnic group), employment, education, mobile phone usage, cigarette smoking, recreational drug usage, self-reported role of alcohol in the injury, mechanism of injury, nature of injury, injury intent, baseline AUDIT, and AUDIT-C mean scores.

#### Analysis of primary outcome

Mixed-effects model for repeated measure (MMRM) method is being used to analyse the primary outcome. The model assesses treatment group, visit, group and visit interaction, the randomisation variables of age, gender, ethnicity and hospital centre (three centres) as fixed effects; baseline AUDIT-C measure as a covariate; and participant as a random effect. The primary outcome is determined by the treatment effect at three months. Variance (co)variance structures are considered to model the within-subject errors, including, but not restricted to, unstructured, compound symmetry, autoregressive AR(1) structures. The Kenward-Roger method is being used to estimate the denominator degrees of freedom for fixed effects. Per Protocol analysis will be performed on the primary outcome as a sensitivity analysis. The per protocol population comprises all randomised participants excluding those who had protocol violations. When appropriate, post-hoc analyses will be conducted.

To assess the effectiveness of the programme for Māori and non-Māori, the analysis of the primary outcome will be repeated with the treatment and ethnicity (Māori vs non-Māori) interaction added to the model. As the study is not powered to test this interaction, the interpretation of this analysis will be made with caution.

#### Analysis of secondary outcomes

A range of self-reported measures of alcohol-related health and social outcomes, and experience with the study, will be evaluated. The difference between the intervention and control groups will be analysed using logistic regression models for binary outcomes adjusting for the randomisation variables of age, sex, hospital centre, ethnicity, and baseline AUDIT-C score.

Differences between the intervention and control groups with respect to time to subsequent injury events (ascertained through probabilistic linkage to national databases) will be evaluated using appropriate survival analytic approaches such as Cox proportional hazards models.

### Methodological limitations

We followed the CONSORT recommendations to design this study. However, given the nature of the intervention, participants are not blind to the intervention. To avoid contamination with information extraneous to the intervention, we did not collect systematic data on treatment adherence through additional questions or prompts during the intervention period. Therefore we cannot assess the number of participants who ignored or deleted the text messages without reading these. However, we can identify the number of participants who sent a ‘STOP’ message to interrupt further messages being sent to them. Furthermore, at the end of the questionnaire administered at the 12-month follow-up phase, we included open-ended questions seeking participants’ responses regarding their experiences, including what they perceived as good things about being in the study and what they liked least.

### Publication and dissemination of results

The results of the trial will be published in peer-reviewed scientific journals with authors meeting the standard criteria for authorship consistent with journal requirements. Study findings and implications for policy and practice will be disseminated in appropriate formats to health service providers, Māori and Pacific communities, other relevant patient groups and agencies, government departments, policy makers, and researchers.

## Discussion

An effective m-health intervention has the potential to overcome some of the commonest barriers to addressing alcohol problems identified in the trauma care setting. This protocol provides detailed information regarding the design and methods of a randomised controlled trial that aims to evaluate the effectiveness of YourCall™, an SMS-text message intervention to support injured patients reduce harmful drinking patterns. The mHealth intervention approach has particular appeal in this context given the high-degree of scalability at relatively low cost per person and the ability to reach low-income groups at least partly mitigating risks of inequitable access to care and support services in the post-injury rehabilitation phase.

Acknowledging the structural determinants that influence and sustain inequities in alcohol consumption and related harm in many populations [24], we also aim to assess the equivalence of the effectiveness of the intervention among Māori and non-Māori participants in the trial. There is an increasing body of research attesting to the possibility that some alcohol-related interventions may inadvertently increase inequities in alcohol consumption and alcohol-related health outcomes [24].

Although the YourCall™ intervention lacks the ‘face to face’ interactivity of traditional brief interventions using motivational interview techniques, it incorporates other attributes that can enhance effective communication, e.g., confidential, personalised and tailored text messages which can be reinforced at periodic intervals, simulating booster interactions [28, 43–45]. A recent review of standard brief interventions for young adults in emergency department suggests that at least one therapeutic contact some distance from the event is associated with more successful outcomes [45].

If found to be effective, linking the YourCall™ intervention to existing services will accrue additional benefits in terms of economies of scale. This can involve the augmentation of community-based services for people with substance use problems and adding a text message option within the Alcohol and Drug Helpline - a nationally implemented information, referral and intervention service. The trial has elicited strong interest and engagement of service providers as contributors in the intervention development team and as members of the study advisory group [22, 35]. Opportunities to translate research to practice are strengthened by the research partnerships with senior emergency and trauma service clinicians. These co-investigators are eager for solutions that can overcome barriers to implementing effective interventions (e.g., workload and resource constraints).

The input of service providers is complemented by the detailed feedback collected directly from patients through open-ended questions exploring their experiences in the 12-month (final) follow-up interview. We expect the information gathered to be of particular importance when considering optimal ways in which to apply the knowledge gained from this study in clinical and community settings.

In conclusion, an estimated seven billion people (95% of the world population) were living in areas covered by a mobile cellular network in 2016 [46]. Not surprisingly, the mobile phone is referred to by some as ‘the most accessible form of mediated communication in world history’ [28]. Capitalising on this ubiquity, text message interventions are becoming increasingly popular as an accessible low-cost mode to nudge behavioural change, including in low- and middle-income countries [45]. However, these strategies do not operate in a vacuum. Good tobacco control policies in high-income countries may account, at least partly, to the success of text message interventions promoting smoking cessation in these settings [47]. In contrast to tobacco control, many countries have weak alcohol harm minimisation policies. In New Zealand, the ubiquitous promotion, availability, and ‘unbridled commercialisation’ of alcohol have raised significant concern [7]. Alongside efforts to address inadequacies in current public policies, text-message interventions can strengthen the range of options available for high risk groups, such as those injured.

## Abbreviations

ACC: Accident Compensation Corporation
AR: autoregressive (structures in models)
AUDIT: Alcohol Use Disorders Identification Test
BI: Brief Intervention
GENACIS: Gender, Alcohol and Culture – An International Study
mHealth: mobile health
MMRM: Mixed-effects model for repeated measures
NI: Named investigator
NIHI: National Institute for Health Innovation
SMS: Short Message Service
